# Genome-Wide High-Resolution aCGH Analysis of Gestational Choriocarcinomas

**DOI:** 10.1371/journal.pone.0029426

**Published:** 2012-01-09

**Authors:** Henriette Poaty, Philippe Coullin, Jean Félix Peko, Philippe Dessen, Ange Lucien Diatta, Alexander Valent, Eric Leguern, Sophie Prévot, Charles Gombé-Mbalawa, Jean-Jacques Candelier, Jean-Yves Picard, Alain Bernheim

**Affiliations:** 1 INSERM U985, Institut Gustave Roussy, Villejuif, France; 2 Université Paris XI, Paris Sud, Orsay, France; 3 INSERM U782, Endocrinologie et génétique de la reproduction et du développement, Clamart, France; 4 Service de carcinologie, service d'anatomie et de pathologie, CHU Brazzaville, Congo; 5 Laboratoire de cytogénétique et de la reproduction, service d'obstétrique, Hôpital A. Le Dantec, Dakar, Sénégal; 6 Molecular Pathology, Institut Gustave Roussy, Villejuif, France; 7 UF de neurogénétique moléculaire et cellulaire, Hôpital de la Salpêtrière, Paris, France; University of Texas MD Anderson Cancer Center, United States of America

## Abstract

Eleven samples of DNA from choriocarcinomas were studied by high resolution CGH-array 244 K. They were studied after histopathological confirmation of the diagnosis, of the androgenic etiology and after a microsatellite marker analysis confirming the absence of contamination of tumor DNA from maternal DNA. Three cell lines, BeWo, JAR, JEG were also studied by this high resolution pangenomic technique. According to aCGH analysis, the *de novo* choriocarcinomas exhibited simple chromosomal rearrangements or normal profiles. The cell lines showed various and complex chromosomal aberrations. 23 Minimal Critical Regions were defined that allowed us to list the genes that were potentially implicated. Among them, unusually high numbers of microRNA clusters and imprinted genes were observed.

## Introduction

Choriocarcinoma results from the malignant transformation of trophoblast cells. They are of interest because allogenic trophoblasts engage in “controlled invasion” of the placenta as part of the normal process of implantation. The numerous syncytiotrophoblastic cells which are deported to the maternal circulation daily do not ordinarily produce metastasis. Conversely, when a trophoblastic malignancy develops, the uncontrolled allografted trophoblast continues to invade and grow anarchically, eventually metastasizing and ultimately leading to death, often due to lung and/or brain metastases. Choriocarcinomas which are, therefore, unique in this respect, could result from normal or complicated pregnancies but half of them arise following a molar pregnancy.

A molar pregnancy is characterized by hydropic degeneration of the placenta [1] due to various causes : diploid androgenesis, with only a paternal chromosomic contribution [2] leading to androgenic complete moles (anCHM); androgenic triploidy with two paternal and one maternal chromosome sets leading to partial moles (PHM) [3]; to a lesser extent with a normal bi-parental contribution, but associated with a maternal NLRP7 mutation, leading to all types of moles (bi-PHM and bi-CHM) [4], [5]. The initial imbalanced expression of genes subjected exclusively to paternal imprinting in anCHM, leads to anarchic dysregulation of numerous other genes. This results in the single abnormal development of a trophoblast without an embryo.

Hydatiform moles (HM), mainly anCHM, could become invasive and malignant once transformed into choriocarcinomas. An incidence of post-molar choriocarcinoma attaining 5% and 18.6% was reported respectively in Senegal [6], [7] and in the Congo [8]. However, it may be higher if we consider a work in which 15 of the 54 moles (about 28%) collected for their gene expression studies became neoplastic [9], [10]. The prognosis of post-molar choriocarcinomas is very heterogeneous, from spontaneous regression, cure or remission under chemotherapy to lethality generally following lung or brain metastases. For example, morbidity due to choriocarcinomas is estimated at about 50% in Senegal [6], [7] and in the Congo [8].

It is still very difficult to determine which molar pregnancies are likely to develop into choriocarcinoma. As these tumors and particularly post-anCHM lesions can be considered as transplanted cancers, immunological factors such as HLA compatibilities [11] could play a role.

What are the genetic events that lead to progression from anCHM, which have a diploid karyotype (90% 46,XX, and 10% 46,XY), to choriocarcinomas? As most human tumors exhibit abnormal karyotypes, which play a major role in malignant transformation, progression and invasion, the study of chromosomal abnormalities in choriocarcinomas could enable us to determine minimal critical regions in this tumor. Using array comparative genomic hybridization (aCGH), a highly resolutive method, the purpose of the present research was to study possible acquisitions of chromosomal abnormalities in choriocarcinomas. The recurrent abnormalities will be associated with candidate genes.

## Materials and Methods

The samples were completely anonymous. The study was a retrospective work, according to a specific protocol approved by the CHU of Brazzaville (Congo) Ethics Committee. The patients gave their informed consent in accordance with the regulations in the Declaration of Helsinki. The data were analyzed anonymously. The Ethics Committee was perfectly aware of the retrospective nature of this work, and of the fact that oral informed consent was obtained for all cases.

Tumor tissue was obtained from 11 post-molar choriocarcinomas : M26, M27, M102, M123, M131 M165, M170, M176, M181, M232, M235 (table 1). All were of uterine origin with the exception of M27 which was a vaginal metastasis from patient M26. The primary tumors were snap frozen in the pathology laboratories. The diagnosis was confirmed by histopathology. All samples exhibited more than 80% of tumor cells.

**Table 1:** Generalities.

Sample	Type(a)	Etiology(b)	Chromosomal aberrations
26	CK, p	U	ND
27	CK, m	U	ND
102,123	CK, p	AD or B	ND
131, 165, 170,	CK, p	A	ND
176, 181, 235,			
232			
BeWo	CK, cl	B	71–72,XXY,add(X)(p21–22),+1,add(1)(p36),+2,−4,+5,−6,add(7)(p22),−11,−13×2,
			i(13q),−18, der(19)t(9;19)(q13;p13),+mar[cps16]
JEG	CK, cl	B	71–74,XXY,add(X)(p21–22),+2,t(4;11)(p15;q13),+5,add(7)(p22),add(7)(q32),+9,t(10;15)(p10;q10),
			t(10;15)(p10;q10),−11,−13,i(13q),−18,del(18)(q22q),+mar[cps21]
JAR	CK, cl	B	66–68,XXY,add(X)(p22),del(1)(p31),t(1;13)(p13;q14),t(3;3)(q12;qter),add(4)(q22),+5,
			−8,del(8)(q22),−10,add(11)(q10),der(13)(q11q34),add(17)(p12),−19,−21,+mar[cps18]

(a) CK, p = primary choriocarcinoma; CK, vm = vaginal metastasis from choriocarcinoma; CK, cl = choriocarcinoma cell line.

(b) A = andromonospermic, AD = , B = , U =  unknown.

Three cell lines established from human choriocarcinoma, BeWo, JEG-3 (named here JEG) [12] and JAR were included in the study. Wo had been grafted from a human cerebral metastasis to the cheek pouch of the hamster, and then serially transplanted [13]. BeWo was isolated *in vitro* from Wo 10 years later [14]. JEG-1 to 8 had been isolated from the transplanted choriocarcinoma in various conditions [12]. JEG-3 is a clone that retained the capacity to synthesize human chorionic gonadotrophin, HCG [15]. JAR was obtained from a placenta tumor grafted by Hertz [13], [16], [17]. Cytogenetic studies were performed on metaphases derived from these cells and chromosome identification was based on R banding. Genomic DNA was extracted from frozen tumors or cell lines using the Qiagen protocol.

### Microsatellite marker analysis

Designed to identify cells of androgenetic origin and to exclude maternal contaminated DNA, the polymorphism of 11 microsatellites (msat) from 3 to 6 chromosomes was determined in most cases as previously described [18]. The same analysis was performed for the three cell lines.

### Fluorescence in situ hybridization (FISH) studies

A set of commercial probes was used to search for abnormalities in chromosome regions : Xq10 (DXZ1) & Yq10 (DYZ1), 14q32 (IGH) & 16q23(MAF), 16q22.1 (CFBF), 18q21 (BCL2, SERPINB2) from Abbott/Vysis ; 12p13.2 (ETV6) from Kreatech and 12q10 (D12Z1) & 12q15 (MDM2) from Zytovision. They were used according to the manufacturer's protocol on histological sections (M176, and M232) or cytogenetic spreads (BeWo, JAR, JEG). A hundred cells were analyzed when available.

### Immunohistochemistry (IHC)

The IHC-P protocol from the Kit Novolink Polymer™ detection System (Novocastra) was used to detect MMP2 (mab 1/100 MAB13407 Chemicon International. Temecula, CA, USA) localized on 16q12.2, and SERPINB2 (rabbit polyclonal 1/600 HPA015480 SIGMA, F), localized on 18q21.3. In all cases (M102, M123, M131, M165, M170, M176, M181, M232, M235), epitope retrieval was performed in 10 mM pH 6 citrate buffer for 45 minutes at 96°C. After the usual steps, slides were incubated with primary antibody for 1 hour at room temperature for the antibodies. After PBS standard rinsing, a post primary block followed by a Novolink polymer were applied for 30 mn each, followed by two series of PBS×1 washing, by DAB revelation and by hematoxylin counterstaining. Negative controls were performed using no primary antibody.

### High resolution aCGH 244,000 K

The choriocarcinomas and the cell lines were analyzed using 244 K microarrays (Agilent Technologies, Santa Clara, CA, USA, G4411B). In all experiments, DNA from pooled human female individuals (Promega, Madison, WI) was used as the reference, with the exception of sample 123 where male DNA was used. Oligonucleotide aCGH processing was performed as specified in the manufacturer's protocol (version 4.0; agilent.com). Data were extracted from scanned images using feature extraction software (version A.9.5.3.1, Agilent). Raw data text files from the latter were then imported for analysis into CGH Analytics 3.4.40. Aberrations were detected with the ADM2 algorithm and filtering options of a minimum of 5 probes and abs (log2Ratio) 0.3. Aberration segments were individually reviewed using build 36, hg18 of UCSC [19]. Abnormalities that were localized to regions with high-copy repetitive or GC-rich DNA sequences including telomeric and centromeric regions were excluded. Copy number abnormalities (CNAs) and copy number variations (CNVs) represented by regions of gains and losses were defined as a linear ratio of ≤0.82 or ≥1.18 respectively with at least 5 oligonucleotides.

Minimal critical regions (MCRs) were defined when similar abnormalities were at least present in two samples. Genes of interest within these MCRs were selected according to at least one of the following criteria: expression in placenta, implication in apoptosis, in cancer, in embryogenesis and parental imprinting. The data are described in accordance with MIAME guidelines and have been deposited in ArrayExpress under accession number E-nTABM-1117.

## Results

### Microsatellite profiling

**Figure 1:**
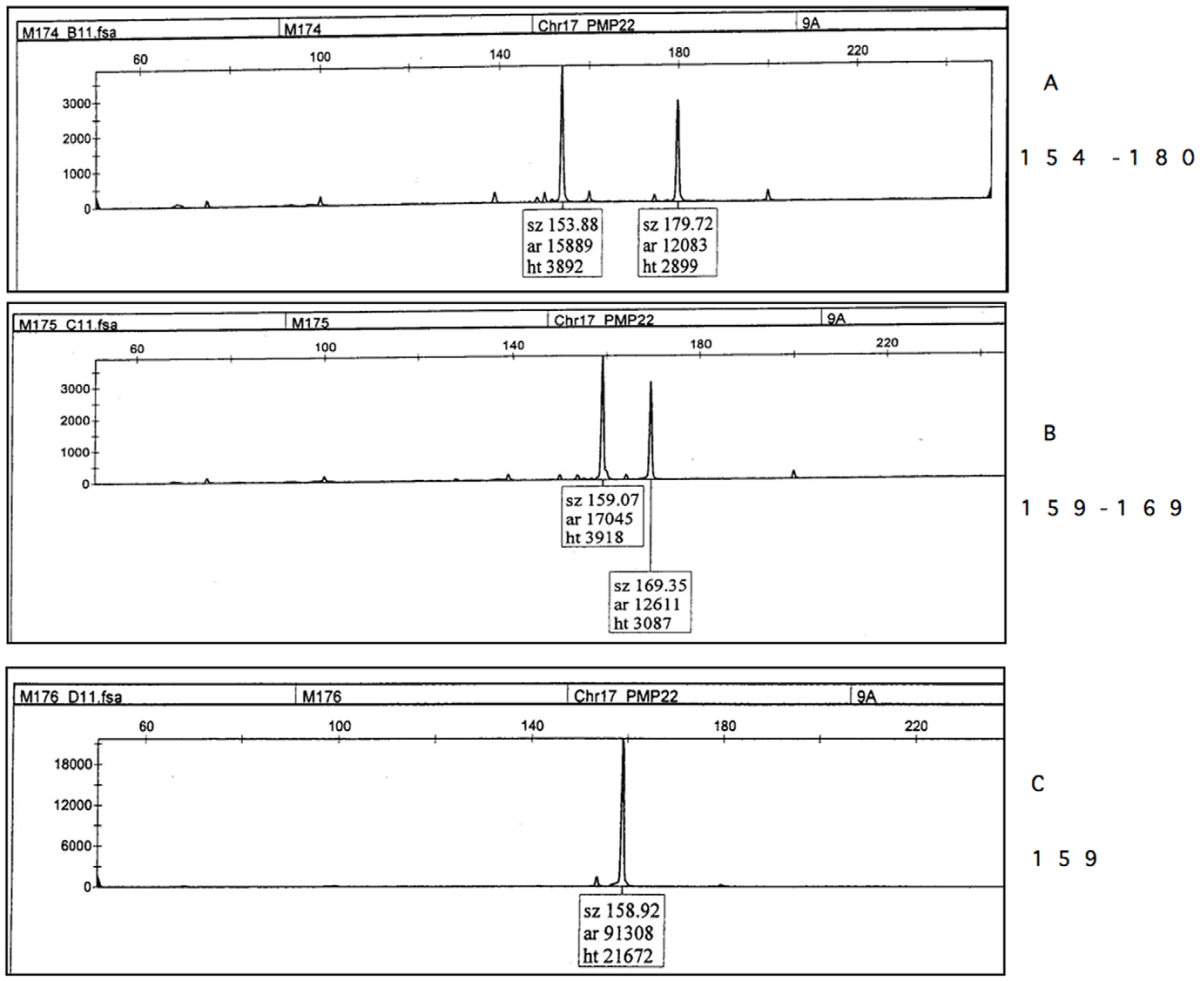
Genotype profile obtained with an automated sequencing apparatus of choriocarcinoma M176 with msat 9A from the PMP22 locus on 17p. [**18**] **.** A Patient/mother genotype with a 154 bp and a 180 bp 9 A alleles ; B father genotype with a 159 bp and a 169 bp 9 A alleles; C Tumor M176 : only a single 159 bp paternal allele is detected. Its absence in the mother allowed us to conclude that it is an andromonospermic tumor; sz = size in base pairs; ar = area; ht = height.

Seven choriocarcinomas out of 9 informative cases (table 1) had a monospermic androgenic origin by microsatellite genotyping (figure 1). The origin of the choriocarcinomas M102 and M123 with heterozygous microsatellite markers was either androgenic, dispermic or bi-parental. The three cell lines had heterozygous markers that were interpreted as a biparental origin. It was compatible with their origin from normal gestations. BeWo and JEG had the same alleles confirming the relationship of the two cell lines, while JAR had different alleles showing an independent origin.

### Cytogenetics

BeWo, JEG and JAR all had a paratriploid karyotype with consistent deletion of the distal part of the X chromosome. Although these cell lines were subjected to multiple passages in culture, the results are compatible with previous reports. The full karyotypes are reported in table 1. JEG had a karyotype marginally resembling that of BeWo (http://www.dsmz.de/human_and_animal_cell_lines/main.php?menu_id=2).

### Array-CGH

#### CNVs

As no matched pairs of normal DNA had been obtained, CNVs [20] were distinguished from CNAs based on various criteria [21] : i) sequence size measuring less than 2 Mb; ii) consultation of the Database of Genomic Variants (www.projects.tcag.ca/variation/) [22]; iii) the type of embedded genes such as olfactory receptor genes, the NF1P1 locus or GSTT1 on chromosome 22 and iv) the presence of repetitive identical breakpoints between patients. Mendelian CNVs, that could harbor coding genes, including microRNA (miRNA), were scattered throughout the entire genome [20], [23] in the choriocarcinoma samples and cell lines. Some of them, for example, UGT2B17 or ADAM5P exhibited high copy number polymorphism within the human lineage (table S1) [23]. As a relationship has been suggested between some CNVs and malignancy [24], several of them are listed in table S1. For example a GSTT gene cluster was reported to be a susceptibility co-factor in cancer [24], [25], [26]. It is noteworthy that a miRNA, i.e. mir-1268, possibly exerting a multigene regulatory effect [27], was found embedded in 15q11.2 CNVs.

#### CNAs

**Figure 2:**
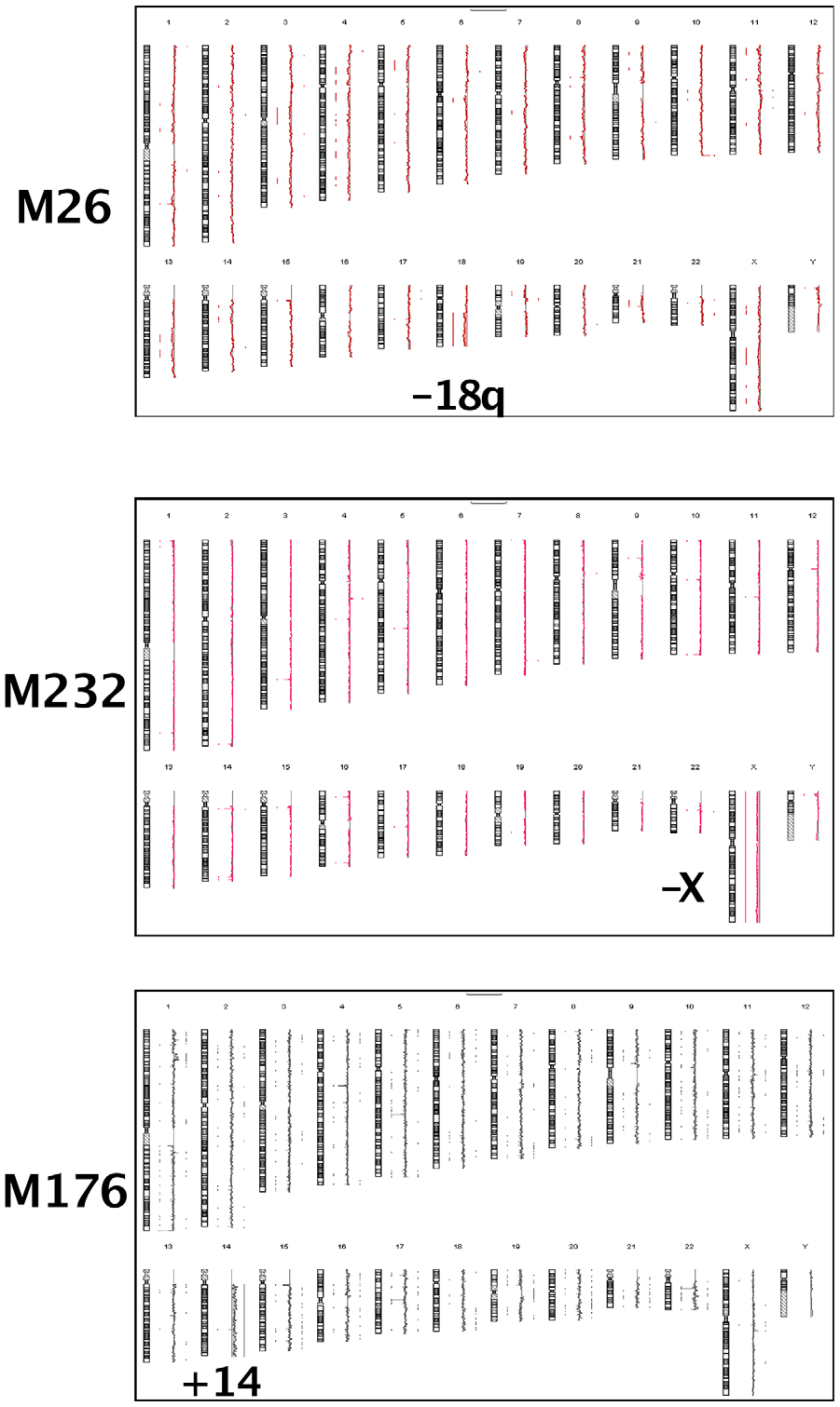
aCGH choriocarcinomas M26, M176, M232. Respectively, loss of 18q, gain of 14q and loss of X. The vertical lines along the chromosomes indicate losses on the left, gains on the right side of chromosomes.

**Figure 3:**
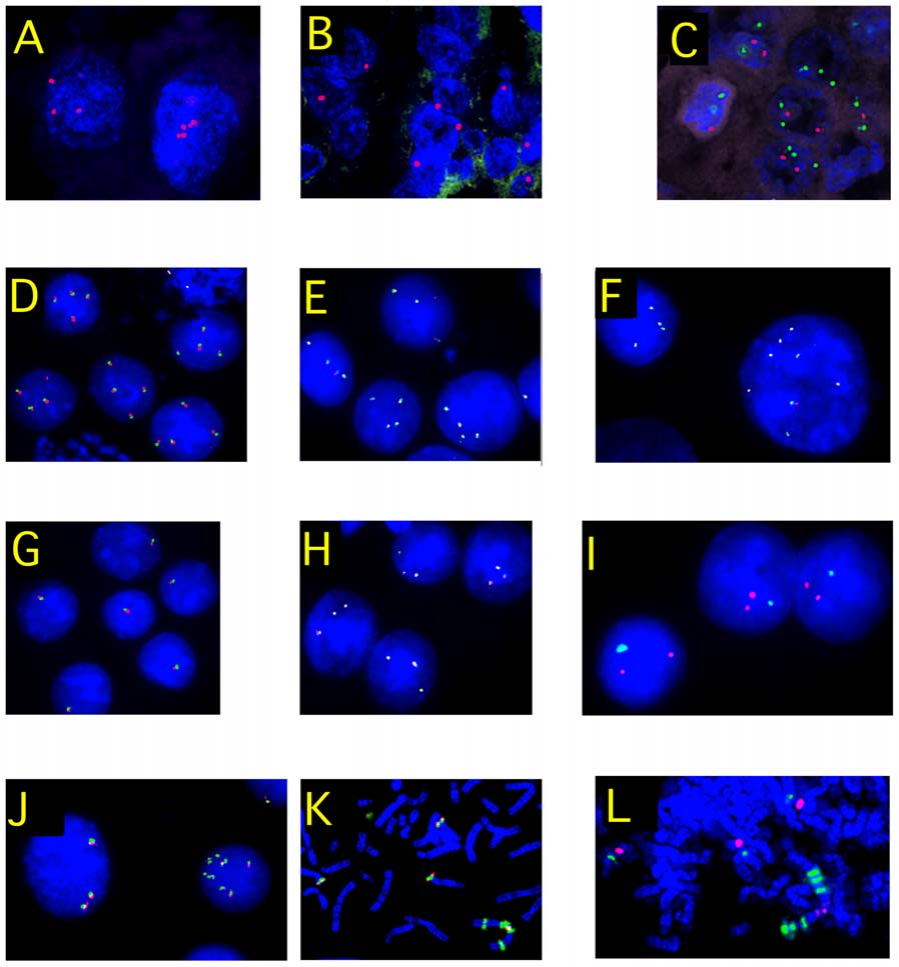
FISH on choriocarcinomas. A = M176 Xq10 r & Yq10 g : left nucleus with 3Xq10, right ones with 5 spots. B = M232 Xq10 r & Yq10 g : only one Xq10 spot for each cell. C = M176 14q32 g & 16q23 r : 2 to 3 green spots (14q) and 1 to 2 red ones(16q). D = JAR 16q22 CBFB r+g : 3 to 4 spots for 16q. E = JEG 16q22 CBFB r+g : 4 spots for 16q. F = BEWO 16q22 CBFB r+g: 4 spots for 16q and a cell with eigth spots; these two types of cells are not distinct by aCGH. G = JEG BCL2 r+g : only one spot of 18q. H = BeWo BCL2 r+g : three spots of BCL2 per cell, that are considered as a small loss compared to JEG. I = BeWo Xq10 r & Yq10 g : two Xq10 and one Yq10 spots. J & K = JAR 12p12 ETV6 r+g : ETV6 is amplified ×8 in the right nucleus of picture J and, on picture K, this amplification is located on a marker chromosome while 3 copies are sitting on probably normal 12p. L = JAR 12q10 r & 12q15 MDM2 g. An amplification of MDM2 is observed (green) from 6 copy on a der(12) with one (or 2) chromosome 12 centromere(s); 3 copies of MDM2 seem present on apparently normal chromosomes 12.

Among the 10 primary choriocarcinomas, M26, M123, M176, and M232 exhibited 20 CNAs, with one case exhibiting 11 CNAs (table S2, figure 2). The chromosomes involved were 1, 11, 14, 17, 18, 19, 20 and X. Loss of the X chromosome was detected in case 232 which showed, by FISH, a single X chromosome without Y in all analyzed cells. In M176 cells, the gain of chromosome 14 was confirmed by FISH with an IGH probe that showed three copies of 14q32 in 42% of cells, while two chromosomes 16 (MAF probe) were observed. A small clone (5%) was detected with four to five chromosomes 14 and three chromosomes 16 (table 2, figure 3). The loss of 18q exhibited by the primary choriocarcinoma M26, was not observed in M27, a metastasis from M26 (table S1). Six primary choriocarcinomas, M102, M131, M165, M170, M181, M235 had no detectable acquired CNA according to aCGH 244 K.

**Table 2:** FISH studies in cell lines.

Localization	Probes and number of signals	Conclusion*	JAR**	BEWO**	JEG**
Xq10 (DXZ1), Yq10 (DYZ1)	DXZ1x2,DYZ1x0	XX			20
	DXZ1x2,DYZ1x1	XXY	65	30	80
	DXZ1x2,DYZ1x2	XXYY		60	
	DXZ1x4,DYZ1x2	XXXXYY	20		
	others		15	10	
16q22.1 (CFBF)	CBFBx3	16q22x3	10		
	CBFBx4	16q22x4	60	72	85
	CBFBx5–6	16q22x5–6		16	
	CBFBx8–10	16q22x8–10	20	12	15
	CBFBx10–14	16q22x10–14	10		
18q21 (BCL2, SERPINB2)	BCL2x1	18q21x1	ND		88
	BCL2x2	18q21x2			12
	BCL2x3	18q21x3		75	
	BCL2x4	18q21x4		5	
	BCL2x5–6	18q21x5–6		15	
	BCL2x10–14	18q21x10–14		5	
12p13.2 (ETV6)	ETV6x3	12px3	10	ND	ND
	ETV6x11–14	12p amp	65		
	ETV6x22–26	12p amp	25		
12q10 (D12Z1), 12q15 (MDM2)	D12Z1x3–4, MDM2>20	12q15 amp	46	ND	ND
	D12Z1x5–6, MDM2>20	12q15 amp	34		
	D12Z1x8–12, MDM2>20	12q15 amp	20		

*The number of X or Y is based from the centromere number.

**Numbers of cells out 100 cells counted.

**Figure 4:**
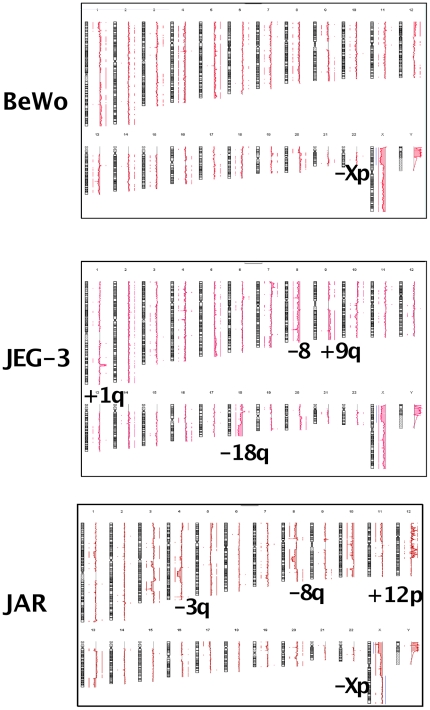
aCGH choriocarcinoma cell lines BeWo, JEG, JAR. Recurrent gain of 5q, 12p, 14q, 20q and loss of 8, 18q, Xp. The CNAs following optimal normalization for the X (see results) are indicated in blue along the X chromosomes in the three cell lines.

The cell lines had multiple unbalanced chromosomal abnormalities with 47 CNAs in JAR, 26 CNAs in BeWo and 31 CNAs in JEG (table S2, figure 4); the all had Y chromosomes, as detected on the aCGH profiles and confirmed by FISH (table 2). The fact that the control DNA was female did not allow us to estimate the number of Y chromosomes by aCGH. The interpretation of the X chromosome ratio also had to take this fact into account.

BeWo and JEG shared exactly the same aCGH deletion on the X chromosome, a 1.3 Mb gain of 5q35.1q35.2 (table S2), a moderate gain of 16 that was confirmed by FISH with a CBFB probe (16q22.1) with 4 copies in most cells (table 2). However, JEG had additional chromosomal abnormalities compared to BeWo, such as an amplification at 1q32.1 (table S2). The peak was 1.36 Mb long with a 4.62 ratio. Another major rearrangement of JEG was the lost distal half of the long arm of chromosome 18 (with only a single complete chromosome left). Loss of BCL2 (18q21.3) was found by FISH in JEG confirming the aCGH data. Three spots were found in BeWo (Table 2), in accordance with aCGH that showed a loss of 18q beginning at 0.7 Mb telomeric to BCL2.

JAR had a complex pattern of chromosomal amplification from 12pter to 12q21.3. Among the numerous genes contained in these amplicons, probes from ETV6 on 12p and MDM2 on 12q were strongly amplified as HSRs that contributed to a marker chromosome (Table 2, figure 3).

#### MCRs

MCRs were built from overlapping of at least two CNAs from different CK or cell lines. The genes had been selected according to the stringent rules described previously and are reported in table 3.

**Table 3:** Minimal critical region (MCR) in choriocarcinomas.

Cytogenetic	Samples	Gain	Width	Localization	Nb of	Selected genes(3)	miRNA
limits(1)	(2)	Loss	(Mb)	(Mb)	genes		
**+1p36.2p36.1**	123 176	G	10.34	8.72–19.06	>100	**PDPN** PAX7	mir-34a
**+1p35.3p34.3**	176 A	G	9.43	35.41–44.84	>100	STK40 CSF3R MYCL1	mir-30e, 30c1
**+1q21.2**	123 A C	G	0.8	148.34–149.23	>20	**CTSK MCL1**	mir-554
**+1q21.3**	123 B A	G	0,34	151,68–152,02	16	S100A1 S100A2 S100A3 **S100A4**	
	C						
+1q32.1	A C(4)	G	4.56	198.64–203.20	>15	**ELF3** PTPN7 MDM4 SOX13 KISS1 FMOD	mir-1231
**+1q42.11q44**	123 A	G	11.29	223.65–234.94	>33	NLRP3 NID1	mir-1182, 1537
+5p15.3p14.2	A B C	G	14.78	0.09–14.79	>29	**TERT**	
+5p13.3p12	A B C	G	31.16	12.87–44.03	>90	**PDZD2**	mir-887, 579, 580, 1274a
+5q23.2q35.2	A B C	G	52.79	121.69–174.48	>100	TGFBI PPP2CA **WNT8A** CD14 GM2A **CSF1R**	mir-1289-2, 886, 874, 584
						**PDGFRB** ABLIM3 **STK10** DUSP1 FGF18 **NKX2-5**	mir-143, 145, 378, 1294, 1303
						HMP19 HLC4 **CLTB** RAB24 **DDX41 MSX2**	mir-146a, 103-1, 103-1-as, 218-2
							mir-585, 218-2, 585
+5q35.2q35.3	A C(4)	G	6.12	174.50–180.62	>75	**MGAT1 BTNL3 hCG_18385**	mir-1271, 1229, 340
−7q32.3q34	B C	L	6.78	132.04–138.80	>20	**CREB3L2**	mir-490
−8p23.3p12	A B C	L	35.68	7.25–36.32	>100	**CTSB DLC1 TUSC3** LPL DUSP4 NRG1	mir-320a, 548i-3, 597, 124-1
						TNFRSF10B RBPMS DUSP26	mir-1322, 598, 383, 320a, 548h-4
−8q13.1q22.1	A B C	L	20.20	66.29–96.49	>100	MMP16 E2F5 TP53INP WDR21C	
						RBM35A RUNXT1	
+9q32q33.1	B C	G	30.07	85.20–115.25	>50	**PAPPA** TNFSF8	mir-199b, 219-b mi
+9q32q34.3	A B C	G	24.04	115.26-139.30	>100	RAPGEF1 ENG CDK9 TRAFF2 PAEP	
−10q22.3q23.2	A B C	L	8.71	79.96–88.67	>35	**ZMIZ1**	mir-126
−11p14.3p12	A B	L	20.69	21.72–42.41	>30	CCDC34 **LGR4 WT1**(p) **GAS2 ELF4**	mir-346
**+11q13.1**	26 27 123	G	2.08	63.47–65.54	100	**RELA MAP3K11** PRDX5 VEGFB CST6	mir-1237, 192, 194-2, 612
+12p13.2p13.1	A B(5)	G	1.55	11.72–13.28	>15	CDKN1B CREBL2 EMP1 **ETV6** **LRP6 DDX47**	mir-1244, 613, 614
						BCL2L14 **DUSP16**	
+12p12.3	A B(5)	G	1.27	15.00–16.27	7	ARHGDIB RERG	
+12p12.3p12.2	A B(5)	G (A)	1.61	18.89–20.50	3	PDE3A **AEBP2**	
+12p12.2p12.1	A B(5)	G	10.79	20.83–31.62	>50	KCNJ8 LDHB RECQL DDX11 FAM60A OVOS2	mir-920
+12q13.1	A B(5)	G	0.34	52.33–52.67	8	ATP5G2 HOXC10 HOXC11 HOXC12 HOXC13	mir-196a-2
**+14q11.2q13.1**	176 A C	G	13.7	19.99–33.69	>100	**BCL2L2 MMP14 EFS**	mir-208a, 208b, 624
**+14q13.1q13.2**	123 176 A C	G	1.33	33.70–35.04	14	**NFKBIA**	
**+14q24.2q24.3**	123 176 A C	G	5.04	72.40–77.45	78	**PGF FOS**	mir-1260
**+14q32.13**	176 A C	G	22.79	77.46–100.25	>50	**SERPINA3 GSC**	mir-342, 345
**+14q32.2q32.3**	123 176 A C	G	5.41	100.26–105.67	>30	**DIO3(p) DLK1(p) RTL(p)** MEG3(m)	**53 mir Rna**
						**ATK1 MTA1 JAG2**	
+16q11.2q24.3	A B C	G	43.64	50.14–88.65	>100	AMFR **CDH5 MMP2** SLC7A5 **MMP15** CSNK2A2	mir-138-2, 328, 1538, 1910, 1972
**+17p11.2**	123 A C	G	1.33	19.12–20.46	>10	MAPK7	mir-1180
**−18q12.2q21.3**	26 C	L	25.5	34.20–59.70	>30	**BCL2 PMAIP1 MBD2 MEDEA TCEB3C(m)** RIT2	mir-924, 1539, 122
**−18q21.3q23**	26 A C	L	25.96	59.70–69.79	>15	**SERPINB2** SOCS6 CDH7 **CDH19**	
**+19p13.1**	26 123 A	G	3.64	16.00–19.64	100	GDF1 UPF1 **INSL3**	mir-640
**+19q13.4**	123 A B(6)	G	0.22	58.76–58.98	2	**DPRX ZNF331(m)**	mir-512-1, 512-2, 1323, 498, 520e
	C					miRna clusters	mir-515-1, 519e, 520f, 515-2, 519c
							mir-1283-1, 520a, 526b, 519b, 525
							mir-523, 518f, 519a-1, 527, 935
							mir-520b, 518b, 526a-1
							mir-520c, 518c, 524, 517a, 519d
							mir-521-2, 520d, 517b
							mir-520g, 516b-2, 526a-2
							mir-518e, 518a-1, 518d, 516b-1
							mir-518a-2, 517c, 520h, 521-1, 522
							mir-516a-1, 1283-2, 516a-2, 519a-2
							mir-371, 372, 373
**+19q13.4**	123 A C	G	1.35	58.99–60.34	3	**NLRP7 NLRP2 PRKCG hCG_2008157**	
**+20q11.2**	123 A B C	G	5.01	29.29–34.30	>80	**DNMT3B(p) PROCR MMP24 BCL2L1** TP53INP2	mir-1825, 644, 499, 1289
**+20q13.2**	123 A B C	G	2.39	49.7–52.09	7	TSHZ2	
**−Xp22.3**	232 A B C	L	3.05	24.8419–7.24	>15		
**−Xp22.31**	232 A B C	L	0.12	7.25–7.37	1	**STS**	
**−Xp22.2p21.3**	232 A B C	L	22.2	7.38–29.86	>15 50	**PRDX4 CNKSR2**	mir-651, 1308

(1) The localization of MCRs with a contribution of de novo choriocarcinomas are indicated in bold.

(2) BeWo = A, JAR = B, JEG = C.

(3) The genes that meet the selection criteria and were selected are indicated in bold characters. The genes subject to parental imprinting are underlined : (m) = maternal imprinted gene; (p) = paternal imprinted gene. The genes that does not meet the selection criteria but are of interest for various reasons are indicated in plain characters.

(4) BeWo and JEG are included for simplicity although they don't define a real MCR.

(5) indicate the “wave-crest” amplification of the JAR cell line probably the site of chromothripsis phenomenon [45]. A basal gain under it was observed between 12pter and 12q21.3.

(6) JAR contribute for only mir 371 372 & 373.

### Immunohistochemistry (IHC)

**Figure 5:**
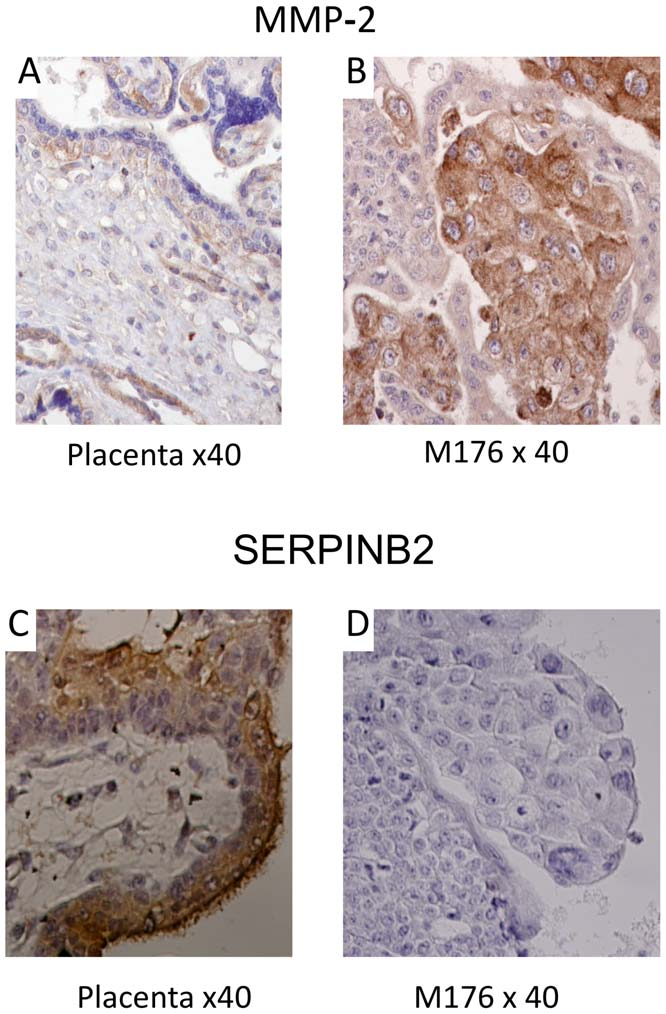
Representative images of IHC. **MMP-2**: (A) Normal placenta 12 weeks = no labeling. (B) CK M176 = strong cytoplasm and membrane labeling of the of CTB cells and MEC. **SERPINB2**: C) Placenta 9 weeks = immunostaining of cytoplasm STB cells and microvillosities. D) CK M176 = no labeling.

Metalloprotein gene (MMP2) expression was tested using IHC in choriocarcinomas and in normal placenta. The cytotrophoblastic (CTB) placental cells were weakly positive, while the choriocarcinoma CTB were heavily labeled (figure 5) in the nine cases examined.

Serpin B2, localized in 18q12, near BCL2, was prominently expressed in syncytiotrophoblasts from normal placenta, although it was not expressed in most choriocarcinomas or very weakly positive in cases 131, 165 and 170 (figure 5).

## Discussion

Six primary choriocarcinomas had no detectable acquired CNA according to aCGH 244 K although they contained more than 80% of tumor cells. Previously observed on metaphasic CGH [28], the situation is reminiscent of Acute Myeloblastic Leukemia where half of the patients have either a balanced translocation and equivalents, or normal karyotypes with some known punctual mutation(s), all of which are undetectable by aCGH [29]. The exclusively paternal genome contribution in choriocarcinomas could enable androgenic homozygozity, that is functionally equivalent to LOH, which would not be detectable by aCGH.

### CNAs

The loss of the X chromosome (M102) and the gain of 14q (M176) were confirmed by FISH (figure 3).

A small gain of 11q13.1 (table S2) was shared by the primary choriocarcinoma M26 and M27, a metastasis from M26. The loss of 18q, exhibited by the primary choriocarcinoma M26, was not observed in M27 suggesting that it was an additional abnormality to the gain of 11q13.1 that occurred after migration of the metastatic clone. Such situations are not infrequent in tumor cytogenetics.

These observations contrasted with the numerous CNAs in cell lines (table S2, figure 4). This is reminiscent of other models such as Burkitt lymphoma where a similar discrepancy exists, although it is less pronounced [21].

The fact that JEG, derived from BeWo [12], shared the same Xp loss at the oligo level, suggests that it was an early chromosomal abnormality. The same observation can be made for the gain of chromosome 16 and the gain of 5q. JEG had additional chromosomal abnormalities compared to BeWo, such as an amplification at 1q32.1 (table S2 and S3). Among numerous genes sitting in this amplicon, ELF3, an ETS family member that has been implicated in cancers and myeloid leukemia cell lines is highly expressed in the placenta [19]; MDM4, an inhibitor of the TP53 gene, has been reported to be amplified in brain tumors [30]. The loss of the distal half of the long arm of chromosome 18 in JEG is quite different of the 18q abnormalities of BEWO. It had its own chromosome abnormalities such as a -7q21.1 not found in JEG, showing an independent chromosomal evolution. This example shows that high resolution array CGH of cell lines could establish a pangenotype and allows one to verify the identity of a cell line or the relationship between cell lines and different subclones, and to follow their evolution through passages.

A progressive deep loss of 8q21.1q22.1, suggesting a highly non random rearrangement, was observed exclusively in JAR (table S3, figure 4). Among multiple genes in this 12.76 Mb region, MMP16 is a placenta-specific metalloproteinase involved in reproduction, embryonic development, and tissue remodeling; TP53INP and WDR21C are apoptosis inhibitors and RUNXT1 is involved in t(8;21) leukemia translocations [31]. JAR also lost 10q25.2q25.3 (table S3), a region that contains TCLF7L2, a HMG gene that is highly expressed in the placenta and is implicated in multiple cancers.

### MCRs

By compiling CNAs in each abnormal sample, we defined 23 MCRs (table 3).

Chromosome 5 was partially gained in the 3 cell lines. Two MCRs contained part of 5p (table 3), that contained the telomerase gene, TERT. A large MCR on 5q (table 3) contained, the proto-oncogene CSF1R (colony stimulating factor 1 receptor precursor) [26], [32] and the growth factor PDGFRB (platelet-derived growth factor receptor), both of which belong to the CSF1/PDGF receptor family of tyrosine-specific protein kinases. CSF1R codes for a cytokine that controls the monocyte-macrophage system and PDGFRB is involved in various myeloid malignancies. These two cytokines are highly expressed in the placenta, control human trophoblast invasion [33] and the EGFR-related family is purported to be critical in the pathogenesis of trophoblastic disease [34]. Also localized in this MCR, STK10 and DUSP1, that shared similarity with a protein 1 kinase domain, had a strong expression in the placenta; CNAs of these genes have been reported to occur in cancer [35], [36]. The distal 5q35.2q35.3 contains the homeobox MSX2, MGAT1 and BTNL3 respectively involved in embryonic development and cancer. All are expressed in the placenta and belong to the HCG family [19].

The -7q32.3q34 MCR (table 3) contained the CREB3L2 gene, a transcription regulator involved in prostate cancer. This region and the BeWo 7q21.1 loss (table S2) did not included the retrotransposon-derived PEG10 gene (paternally expressed imprinted gene 10) whose loss causes early embryonic lethality due to placental defects [37], [38].

Loss of the 8p23.3p12 region, previously reported in primary choriocarcinoma [39], [40], harbored (i) the CTSB gene, a cathepsin implicated in invasion and metastasis and (ii) DLC1 and TUSC3, two tumor suppressor genes that are expressed in the placenta, and often deleted in many cancers.

In JEG and JAR, a large MCR+9q32.2q34.3 contained a very highly expressed metalloproteinase PAPP-A (pregnancy-associated plasma protein-A) which plays a role in cell proliferation, invasion and metastasis [24], [41].

Among the numerous genes present in the 20.7 Mb loss on 11p, common to BeWo and JAR, were LGR4 which plays an important role in female reproduction and in early-stage embryonic development [42] and WT1, the well-known suppressor gene which is implicated in nephroblastoma (Wilms tumor) but also in mesenchymal stem cells tumor [43].

The 11q13.1 gain, exclusively contributed by *de novo* choriocarcinomas, contained RELA, a nuclear transcription factor NF-kappa-B complex subunit. MAP3K11 kinase preferentially activates MAPK8/JNK kinase, and functions as a positive regulator of the JNK signaling pathway; it is found to be involved in the transcription activity of NF-kappaB [19]. This MCR contains 4 miRNA genes (table 3).

BeWo had a gain of an entire 12p arm while JAR had a “wave-crest” amplification appearance spanning 12pter to 12q21.3 that resulted in 10 different regions, exclusively taking into account the summit of the gains. This pattern suggests an extensive breakage-fusion-bridge process [44] together with possible inversions from the chromosome 12 region or even chromothripsis [45]. A large number of genes were implicated in these very complex rearrangements (table 3, figures 3 and 4).

A recurrent gain of 14q was observed in two tumors. M176 had gained a whole chromosome 14, and M123 had partial gains which had also occurred in the BeWo and JEG cell lines. The most centromeric 10.21 Mb MCR contained among numerous genes, EFS (embryonal Fyn-associated substrate isoform 2), that is important in intracellular signal transduction and MMP14 that codes for a matrix metalloproteinase 14 preproprotein. Both genes are highly expressed in placenta. Three miRNA are localized in this region (table 3).

The next 1.33 Mb gained MCR harbored NFKBIA which codes for an inhibitor of the NFKB complex. The +14q21.3q24.3 MCR, harbored FOS (PDGF), a proto-oncogene and PGF (placental growth factor/vascular endothelial growth) that belongs to the PDGF-VEGF family; it is a growth factor with a cell proliferation function in the placenta where it is very highly expressed.

The gain of 14q32.2q32.3, a region subject to parental imprinting, was observed in two tumors and two cell lines. It contained the postfertilization-derived secondary MEG3-DLK1 intergenic differentially methylated region [46]. Three such genes, strongly transcribed in the placenta, are next to MEG3 : DLK1 (Delta-like homologue 1, also known as preadipocyte factor 1), DIO3 (type III iodothyronine deiodinase 3), both containing an EGF-like domain, and RTL1 (retrotransposon-like 1). Their overexpression causes several embryonic defects, retarded growth and lethality [27], [47]. RTL1 is a key gene in placenta formation [48]. It is a retrotransposon-derived, paternally-expressed imprinted gene that has an overlapping maternally expressed antisense transcript. This transcript contains several miRNAs targeting the transcripts of this gene through an RNA interference (RNAi) mechanism. RTL1 is essential for fetal capillaries. Its overproduction is associated with abnormal fetal development in mice. Furthermore, in humans and in mice [49], the DLK1-DIO3-RTL1 imprinted domain contains more than 50 miRNAs that exert multiple interactions with numerous genes including oncogenesis [50], [51]. These results suggest that the biallelic paternal imprinted genes and the multiple miRNAs located in 14q32 in humans and in 12qF1 in mice, could contribute to the pathogenesis of trophoblastic disease [52] and to CK.

The 16q was gained in the three cell lines (table 3). It harbored MMP2, a metalloprotein gene, found to be overexpressed by RT-PCR in the three cell lines and by Western blot in JAR and BeWo [53]. By IHC, choriocarcinoma cytotrophoblastic cells were heavily labeled (figure 5) in the nine *de novo* cases examined, contrasting with the control placental cells that were weakly positive. However, no gain of 16q was detected in primary tumors neither by aCGH nor by FISH (M176). This apparent discordance between IHC expression and DNA copy number is not unusual and among many reasons, can be caused by small duplications that are undetectable by aCGH even at high resolution. An example is the duplication of MLL that is sometimes enhanced by a trisomy 11 in AML [31].

A deep deletion, −18q12.2q21.3, with a 0.37 linear ratio, formed a 26.96 Mb MCR (table 3) that was confirmed by FISH with a BCL2/SERPINB2 probe in JEG with only one copy. This MCR contains also PMAIP1/Noxa, a tumor suppressor gene that contributes to p53-dependent apoptosis after genotoxic exposure [54] and is implicated in various cancers. In BeWo, the CNA breakpoint was between BCL2 and SERPINB2 (Placental plasminogen activator inhibitor). SERPINB2 plays a role in the placenta during early pregnancy at 8–10 weeks [55], is very highly expressed in this tissue [19], is purported to be involved in gestational trophoblastic disease [56] and in malignant neoplastic progression [57]. However it was not found or very poorly expressed in the *de novo* choriocarcinoma studied in the present work (figure 5). The genetic or regulatory origin of this lack of expression remains to be determined.

A 3.64 Mb gain was observed on 19p13.1, with more than a hundred genes encoded in this strong chromosomal R band. Although very small CNVs were present in this small area, no large ones were detected (DGVDB Toronto) [19]. Only a few genes were selected based on their high placental transcription ratio (table 3) [19]. On 19q13.4, a smaller gained MCR (table 3), contained DPRX, an homeobox DNA binding protein with multiple related processed pseudogenes which might be involved in embryonic cells. Two miRNA clusters, one containing more than 50 miRNAs named C19MC [52] and a smaller one miRNAs 371–373 were located within these 0,22Mb. C19MC was found to be paternally imprinted and expressed in placenta, CHM and the JEG cell line [52]. It was found to be overexpressed in multiple tumors including malignant germ cell tumors [58]. Next to this region was a 1.35 Mb gained MCR (table 3) containing the NLRP7 gene involved in endometrial cancer [59] and associated with biparental complete hydatidiform moles [60], [61].

On chromosome 20q11.2, a 5.1 Mb gained MCR contained more than 100 genes. Paternally imprinted DNMT3B encodes a DNA methyltransferase which is thought to function in *de novo* CpG methylation. Its homolog DNMT3A was recently found to be mutated in a subset of leukemia with a normal karyotype [62]. The PROCR (alias EPCR) encodes an endothelial protein C receptor that is a serine protease expressed in placental arteries and veins [63] and is detected in giant trophoblast cells at the feto-maternal boundary in mice. The metalloproteinase MMP24 gene plays a role in fetal losses and embryonic development.

The X chromosome was the most involved in four samples (tabme 3). A complete loss was observed in the primary tumor 232, while a partial loss of Xp was found in the three cell lines which all had two X chromosomes, as depicted by FISH (table 2). JAR also had a 0.12 Mb Xp22.3 deletion of the other X chromosome superimposed on the larger deletion from the other X. It defined the smallest MCR in this region which contained only the 3′ part of the STS (steroid sulfatase) gene. Some CNVs were in the centromeric part of this MCR but almost none of them had extended to the STS coding region. This gene, which is necessary for the production of free steroids from sulfoconjugated precursors, is highly expressed in normal placenta, contributes during pregnancy to the synthesis of estrogen, and is involved in embryonic and fetal development. STS plays an important role in the development and progression of various cancers such as breast carcinoma [25] or estrogen-dependent cancers such as human endometrial carcinoma [64]. Although it is most probably a somatic alteration, it cannot be ruled out that this 120 Kb deletion could also be a constitutional CNA, corresponding to X-linked ichthyosis.

A 30 Mb deletion -Xp22.3p21.3 (table 3), was present on the other X chromosome of JAR defining the Xp MCR that contained more than 25 genes, including STS. The PRDX4 (Peroxiredoxin-4) gene is a transcription factor involved in oocyte maturation and in early embryo development [65] and protects it against oxidative stress [66], [67]. CNKSR2 (connector enhancer of kinase suppressor of RAS) seems to be an antiapoptotic gene and a possible regulator of various pathways including that of RAS [68].

In previous studies using mainly metaphasic comparative genomic hybridization, a gain of 7q [39], [40], [69] has been described in choriocarcinoma although it was not observed in this work. The 8p12–p21 loss observed in the present study, has previously been reported in gestational choriocarcinoma [40] and in non gestational choriocarcinoma [70] where it was associated with gains of 5p, 20q and losses of 18q and Xp. These rearrangements were identified as MCRs in the present work (table 3) with deep losses of 18q, Xp and gains of 5p and 20q. They were not observed in a choriocarcinoma derived from a Non Seminomatous type II Germ Cell Tumor originating in a case of 46,XY gonadal dysgenesis [71]. The presence of a Y chromosome in BeWo JAR JEG cell lines, the derivations of a choriocarcinoma cell line from two XY patients compare to one from a female [17], may reflect a a proliferative advantage for cell lines establisment as the predisposition to malignant transformation in gonadal dysgenesis induced by the Y chromosome [71].

Recurrent MCRs in choriocarcinomas harbor a high number of microRNA clusters and imprinted or paternally expressed miRNAs genes [52] which constitute the uniqueness of these tumors amid most adult cancers. As these chromosomal abnormalities are present in cell lines, this opens the way for future functional studies in these complex malignancies.

## Supporting Information

Table S1 **Copy number variations (CNV).** (1) included in high copy number variations.
(RTF)

Table S2 **Copy number aberrations (CNAs) in choriocarcinoma by aCGH.** (1) M102 was a biparental choriocarcinoma because a Y was detected. As the reference DNA was female, an apparent loss of the X appeared; the background was too high which prohibited detailed analysis. (2) Metastasis from M26. (3) Probably the site of a chromothripsis phenomenon.
(RTF)

Table S3 **Single CNA with genes of interest.** (1) Strong gains or even amplifications.
(RTF)
