# Supplementary material for: Genome-Wide High-Resolution aCGH Analysis of Gestational Choriocarcinomas
Source: PLoS One. 2012 Jan 9;7(1):e29426. doi: 10.1371/journal.pone.0029426 (PMC3253784; doi:10.1371/journal.pone.0029426)
Supplement: Table S1 — Copy number variations (CNV). (1) included in high copy number variations. (RTF) [file pone.0029426.s001.rtf]

Table S1 . Copy number variations (CNV)

Localization	Width (Mb)	Position (Mb)	Nb of genes	 selected gene embeddeds	
+1p36.3	1,42	1.11-2.52	>55	SKI	
1p36.2	0.06	12.78-12.84	4	PRAMEF2	
1q44	0.036	246.82-246.85	2	OR2T10	
4q13.2	0.09	69.06-69.16	1	UGT2B17(1)	
5p15.3	0.07	0.80-0.87	1	ZDHHC11	
7q34	0.7	142.12-142.20	>5 	TRCVB	
8p11.2	0.16	39.34-39.50	2 	ADAM5P(1)	
10q11.2	1.33	46.39-47.73	> 5 	ANXA8L2	
11q11	0.07	55.12-55.19	4 	OR4C11	
14q11.2	2.2	19.26-19.49	6 	OR4K1	
14q11.2	0.5	21.43-22.03	>5 	TCRA	
15q11.2	0.6	18.83-19.46	>5	BCL8, NF1P1, mir-1268	
17q21.3	0.04	41.52-41.56	1 	KIAA1267	
22q11.2	0.47	22.67-22.72	2	GSTT1	

(1) included in high copy number variations 
