# Supplementary material for: Genome-Wide High-Resolution aCGH Analysis of Gestational Choriocarcinomas
Source: PLoS One. 2012 Jan 9;7(1):e29426. doi: 10.1371/journal.pone.0029426 (PMC3253784; doi:10.1371/journal.pone.0029426)
Supplement: Table S2 — Copy number aberrations (CNAs) in choriocarcinoma by aCGH. (1) M102 was a biparental choriocarcinoma because a Y was detected. As the reference DNA was female, an apparent loss of the X appeared; the background was too high which prohibited detailed analysis. (2) Metastasis from M26. (3) Probably the site of a chromothripsis phenomenon. (RTF) [file pone.0029426.s002.rtf]

Table S2. Copy number aberrations (CNAs) in choriocarcinoma  by  aCGHSample	 CNA	Position (Mb), [ratio]	Cell 	 CNA	Position (Mb), [ratio]	
M102(1)	No(1)  	 	JAR	-1p31.1p22.3	 71.46-86.91 [0.72]	
M131	No	 	 	+1q21.3	151.68-152.02 [1.46]	
M165	No	 	 	+3p25.1p22.2	12.56-39.25 [1.6]	
M170	No	 	 	-3p21.31	50.35-50.90 [0.79]	
M181	No	 	 	-3q11.2q13.1	-95.02-108.49 [0.72]	
M235	No		 	+3q13.1q24	112.24-144.79 [1.50]	
M123	+1p36.2p35.3	8.72-29.23 [1.36]	 	-3q24q26.1	146.38-168.35 [0.70]	
	+1p13.2	112,78-113,53[1.23]	 	+4p16.1	6.80-8.98 [1.31]	
	+1q21.1q23.3	143.82-160.02 [1.24]	 	-4p12	 48.18-48.31 [0.74]	
	+1q42.1q43	223.65-234.94 [1.21]	 	-4q25q31.2	111.78-148.25 [0.64]	
	+11q12.2q13.2	60.80-67.13 [1.31]	 	+5p15.3p15.2	0.09-14.79 [1.46]	
	+14q13.1q13.2	 33.70-35.04 [1.27]	 	+5p15.2p12	12.87- 44.03 [1.30]	
	+14q24.2q24.3	 72.40-77.45 [1.20]	 	+5q23.2q35.2	121.69-174.48  [1.25]	
	+14q32.3q32.3	 100.26-105.67 [1.23]	 	-7q31.1q34	110.84-138.80 [0.67]	
	+17		 	-8p23.1p12	7.25-36,32 [0.70]	
	+19		 	- 8q13.1q22.1	66.29-84.72 [0.6]	
	+20q	26.56-35.35 [1.27]	 	- 8q21.1q22.1	84.72-96.49 [0.48]	
M176	+ 1p36.3p36.1	2.53-19.06 [1.24]	 	+8q23.3q24.3	115.85-146.26 [1.25]	
	+1p35.3p34.1	29.56-44.84 [1.7]	 	+9q32q34.3	115.26-139.30 [1.25]	
	- 1q21.2	148.74-148.86 [0.72]	 	-10p15.3q23.2	0.12-88.26 [0.73]	
	+14q	19.38-106.30 [1.5]	 	-10q25.2q25.3	113.91-116.56 [0.51]	
M232	 -X	 	 	-10q25.3qter	116.63-133.66 [0.74]	
M26	+11q13.1	63.41-65.54 [1.25]	 	-11pterp11.2	0.19-46.69 [0.75]	
	-18q12.2q23	34.20-75.22 [0.75]	 	+12p13.3 	0.03-9.81 [1.35] (3)	
	+19p13.11 	16.46-19.64 [1.22]		+12p13.2p13.1	11.72-13.28 [2.3] (3)	
M27(2)	+11q13.1	63.47-65.55 [1.20]	 	+12p12.3 	15.00-16.27 [2.1] (3)	
		 	 	+12p12.3p12.2 	18.89-20.50 [3.6] (3)	
		 	 	+12p12.2p11.2	20.83-31.62 [2.1] (3)	
		 	 	+12q12q13.1	37.37-44.71 [1.3] (3)	
			 	+12q13.1	52.33-52.67 [2.29] (3)	
(1)  M102 was a biparental choriocarcinoma because a Y was detected. As the reference DNA was female, an apparent loss of the X appeared; the background was too high which prohibited detailed analysis. (2) Metastasis from M26(3) Probably the site of a chromothripsis phenomenon.
Table 3 (continued). Copy number aberrations (CNAs) in choriocarcinoma by aCGHCell	 CNA	Position (Mb), [ratio]	Cell	 CNA	Position (Mb), [ratio]	
JAR cont	+12q14.1q15	57.12-70.02 [2] (3)	BeWo	+19p13.1p12	19.76-22.72 [1.24]	
	+12q21.1	71.55-71.90 [2.22] (3)		+19q12	33.73-38.88 [1.21]	
	+12q21.1q21.3	73.98-86.60 [2.3] (3)		+19q13.4	58.96-63.78 [1.27]	
	-13q11q12.13	18.06-24.47 [0.75]		+20p12.1q13.2	15.22--52.09 [1.30]	
	+13q12.1q31.1	24.57-77.85 [1.28]		-Xp22.3p11.3	4.19-42.75 [0.50](4)	
	-13q31.3qter	91.60-113.65 [0.69]	JEG	+1q21.2	148.34-149.23 [1.36]	
	+16q12.1q24.3	50.14-88.69 [1.20]		+1q32.1	198.64-203.20 [3.68]	
	 -17p13.1p12	9.10-12.79  [0.76]		+2.	 	
	-19p13.3p13.1	0.83-17.20 [0.65]		+5p15.3q14.2	0.90-103.61 [1.26]	
	+19q13.4	58.92-58.98 [1.3]		+5q22.2qter	111.73-180.62 [1.56]	
 	+20p13-q11.2	0.01-34.30 [1.20]		+7p22.2p21.2	3.81-14.91 [1.74]	
	-20q12-q13.1	38.16-43.54 [0.75]		+7q31.3q32.1	126.38-128.94 [1.36]	
	+20q13.2-qter	49.7-62.37 [1.24]		-7q32.3qter	132.04-158.76 [0.74]	
	-Xp22.3	2.84-7.24 [0.54](4)		-8	 	
	-Xp22.3	7.25-7.37 [0.1](4)		+9q13qter	.23-140.24- [1.55]	
	-Xp22.2p21.2	7.38-29.97 [0.50](4)	 	+10pterq22.2	0.11-75.62 [1.45]	
	+Xq21.2q28	85.58-151.05[1.30](4)		-10q22.3q23.2	79.96-88.67 [0.69]	
BeWo	-1p36.3p36.1	1.60-24.23 [0.7]		+11p15.1	19.97-21.56 [1.43]	
	+1p34.3-p31.2	35.41-68.72 [1.26]		+12q14.3q15	65.73-66.37[1.31]	
	+1q21.2q44	148.31-247.17 [1.5]		+14.	19.99-105.56 [1.27]	
	-4	 		-15q23q25.1	69.9876.75 [0.77]	
	+5	 		+16q11.2q12.1	45.05-47.21 [1.26]	
	-8	 		+16q12.2q21	54.14-57.56 [1.26]	
	-7q21.1	77.69-89.69 [0.79]		+16q21q24.3	63.86-88.69 [1.35]	
	+9q13qter	85.20-140.24- [1.21]		+17p11.2	19.12-20.46 [1.31]	
	-10q11.2q25.1	52.50-110.98 [0.77]		-18pterp11.	0.12-15.11 [0.70]	
	+11p15.1	19.97-21.56 [1.36]		+18q11.2	16.79-19.22 [1.60]	
	-11p14.3p13	21.72-42.41[0.77]		-18q11.2q21.2	20.61-50.15 [0.65]	
	+12pterp11.1	0.07-34.64 [1.6]		-18q21.2q23	50.15-75.92 [0.37]	
	+12q13.1q14.1	46.38-56.76 [1.31]		+19q13.4 	58.61-60.34 [1.39]	
	+14q11.2q32.3	19.99-105.56 [1.27]		+20q11.2	29.29-32.33 [1.35]	
	+16p112.1p11.2	22.80-31.15 [1.25]		+20q11.2	32.34-34.90 [1.70]	
	+16q11.2q21	45.01-57.75 [1.21]		+20q11.2qter	34.91-62.37 [1.30]	
	+16q21qter	64.35-88.65 [1.3]		+22q11.2	20.14-21.39 [1.32]	
	+17p11.2	  18.81-20.64 [1.25] 		+22q13.3	44.42- 49.56 [1.30]	
	+18q11.2	 17.65-19.13		-Xp22.3p11.3	4.19-42.75 [0.50](4)	
	-18q21.3q22.3	59.70-69.79 [0.76]				
	+19p13.1	16.00-19.72 [1.62]				
(1) M102 was a biparental choriocarcinoma because a Y was detected. As the reference DNA was female, an apparent loss of X appeared. The background was too high which prohibited detailed analysis. (2) Metastasis from M26(3) Probably the site of a chromothripsis phenomenon.(4) The CNA of the X chromosome were extrapolated, as the number of X chromosomes were calculated on the basis of a paratriploid DNA content and a 46,XX control.
