# Supplementary material for: Genome-Wide High-Resolution aCGH Analysis of Gestational Choriocarcinomas
Source: PLoS One. 2012 Jan 9;7(1):e29426. doi: 10.1371/journal.pone.0029426 (PMC3253784; doi:10.1371/journal.pone.0029426)
Supplement: Table S3 — Single CNA with genes of interest. (1) Strong gains or even amplifications. (RTF) [file pone.0029426.s003.rtf]

Table S3. Single CNA with genes of interest

Cytogenetic 	Samples	Gain/	Width	Ratio	Localization 	Nb of 	Selected genes 	miRNA	
Limits  		Loss	(Mb)		(Mb)	genes			
+1q32.1	JEG	G	4,56	3.68 (1)	198.64-203.20		ELF3, MDM4	mir-1231	
-7p11.2	M176	L	0.29	0.77	54.93-55.23	1	EGFR		
+7p15.3p15.1	JEG  	G	7.8	1.3	23.13-30.99	73	 IGF2BP3 HOXA1 HOXA4 	mir-148a, 196b, 550-1	
							 HOXA5 HOXA7 HOXA9 HOXA10 		
-7p12.2p12.1	JAR	L	0.9	0.76	50.57-51.48	<5	GRB10		
-8q21.1q22.1	JAR	L	12,76	0.48	84.72-96.49		MMP16 TP53INP WDR21C RUNXT1		
+10q11.21	JEG	G	3.2	1.25	42.24-45.47	33	RET		
-10q25.2q25.3	JAR	L	2,65	0.51	113.91-116.56		CASP7 ADRB1 TCLF7L2		
-11p15.5p112	JAR	L	46.5	0.75	0.19-46.69	448	H19 ASCL2 KCNQ1 PHLDA2	mir-210, 675, 483, 302e	
							 P57 CDKN1C	mir-610, 129-2	
-12q13.13	JAR	L	0.12	0.78	48.87-49.00	2	LIMA1	mir-1293	
+12q13.2q14.1	BeWo	G	2.35	1.3	54.41-56.76	88	RAB5B  MMP19  IL23A 		
+12q21.2q21.3	JAR	G	1.42	3.9	77.75-79.18	4	SYT1 PAWR 	mir-1252	
-17p13.1p12	JAR	L	3.6	0.76	9.10-12.79	28	MAP2K4 	mir-744	
-17q25.3	M176	L	0.24	0.78	74.34-74.58	7	TIMP2		
-19p13.2p13.1	JAR	L	6,28	0.73	10,91-17,20	168		mir-24-2, 27a, 23a, 181c, 181d, 639	
+20q13.1q13.2	JEG	G	8,82	1.3	41,21-50,03	125	MMP9	mir-1259, 645, 1302-5	

(1) Strong gains or even amplifications 
